# Multi slice DENSE in a single breath hold

**DOI:** 10.1186/1532-429X-11-S1-P221

**Published:** 2009-01-28

**Authors:** Andreas Sigfridsson, Henrik Haraldsson, Tino Ebbers, Shinichi Takase, Hajime Sakuma

**Affiliations:** 1 Mie University, Tsu, Mie, Japan; 2 Linköping University, Linköping, Sweden

**Keywords:** Single Slice, Myocardial Strain, Single Breath Hold, Eulerian Strain, Multiple Breath

## Introduction

Displacement ENcoding with Stimulated Echoes (DENSE) is a method to acquire displacement maps of the myocardial motion. This can in turn be used to estimate strain which is an important measure of myocardial function. Previously, DENSE imaging has primarily been performed using multiple breath holds per slice, severely limiting its incorporation into the tight schedule of clinical routine. Strain maps are further desired throughout the left ventricle, not only in a single slice. A targeted approach, guided by results from perfusion and late enhancement studies is tempting, but the presence of contrast agent shortens the T1 relaxation, which hampers stimulated echo acquisitions such as DENSE. We therefore propose a multi slice acquisition for strain measurements in a single breath hold. We compare this with conventional single slice DENSE acquired in separate breath holds.

## Methods

Ten healthy volunteers were imaged in a Philips Achieva 1.5 T scanner, using the standard 5 channel cardiac coil. Three slices were acquired at the basal, equatorial and apical short axis levels in a single breath hold multi slice acquisition.

End systolic displacement was measured by encoding the position at the R-wave using a SPAMM 1–1 sequence, and decoding at end systole. The appropriate trigger delay time was determined using a long axis cine imaging sequence. The three slices were excited and read out after each other in the same cardiac cycle, with 25 ms separation.

Imaging parameters were: field of view 350 mm, slice thickness 8 mm, matrix 128 × 115, SENSE factor 2, TFE-factor 3, EPI-factor 7, TR 8.9 ms, TE 4.2 ms. Displacement encoding strength was 0.35 cycles per pixel. Flip angle was optimized to yield highest constant signal strength [1].

Both in-plane displacement directions were measured using displacement encoding in three oblique directions [2]. This allows for the subtraction of background phase errors and increases the displacement-to-noise ratio while maintaining encoding strength, and thus signal dephasing. Complementary encoding was used to suppress the T1 relaxation echo[3]. Each direction was acquired in six heart beats, resulting in a total scan time of 18 heart beats.

For comparison, the three slices were also acquired separately in one 18 heart beat breath hold each. Apart from only acquiring a single slice at a time, the scan parameters were identical. The order of the single slice and multi slice acquisitions was randomized.

Eulerian strain was analysed using custom software written in MATLAB. Strain was evaluated in ROIs placed manually according to the AHA 17-segment model with the apical segment excluded.

## Results

**Figure 1:**
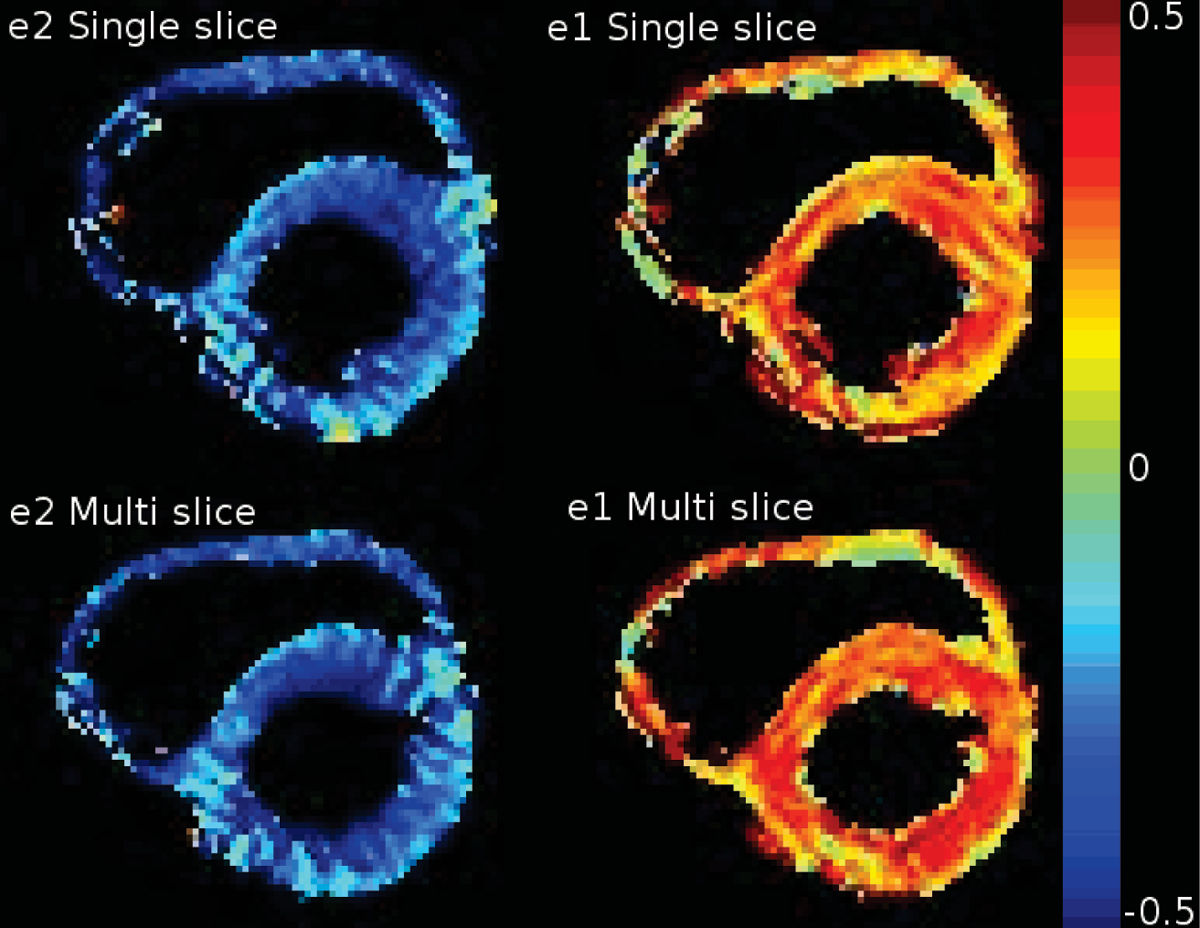
Figure 1

Strain maps of eulerian strain eigenvalues e1 and e2, corresponding to expansion and contraction, are shown in Figure 1 for single slice and multi slice acquisitions of one volunteer.

**Figure 2:**
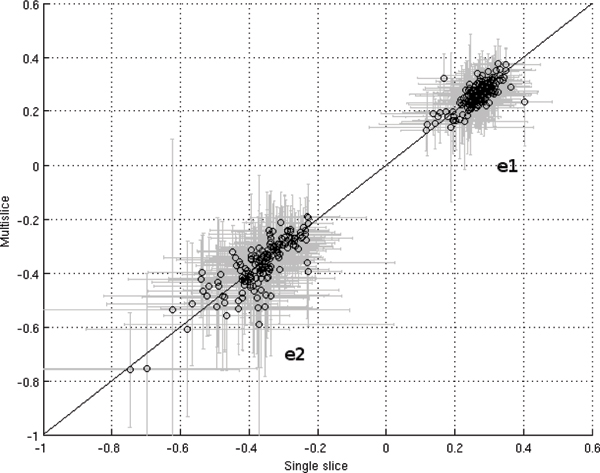
Figure 2

Regional strain values from all volunteers are plotted in Figure 2. Error bars indicate the standard deviation within each ROI.

**Figure 3:**
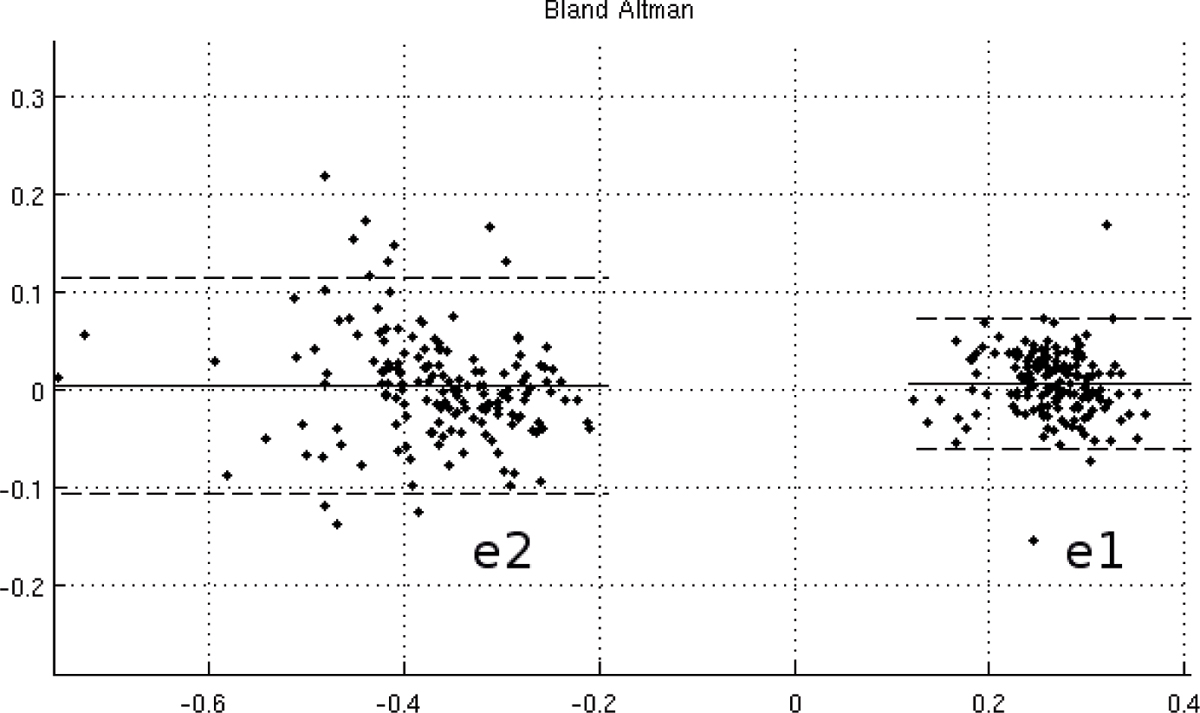
Figure 3

A Bland-Altman analysis is shown in Figure 3. For e2 (contraction), the bias is 0.004 and limits of agreement -0.11 and 0.12. For e1 (expansion), the bias is 0.007 with limits of agreement -0.06 and 0.07.

## Discussion

A method for acquiring myocardial strain in the whole left ventricle in a single breath hold has been presented. It has been shown that acquiring three slices in the same breath hold results in strain values that agree well with those acquired in three separate breath holds. The small differences between the methods indicate that the slight shift in acquisition timing between the slices (25 ms) did not significantly influence the strain values.

Eliminating the need for multiple breath holds, multi slice DENSE extends the utility of DENSE for myocardial strain analysis.

## References

[1] Stuber (1999). MAGMA.

[2] Lin (2008). MRM.

[3] Gilson (2004). MRM.

